# Mechanism of action of the sinus microbiome in postoperative recurrence of type 2 chronic rhinosinusitis and advances in targeted intervention

**DOI:** 10.3389/fimmu.2026.1889314

**Published:** 2026-08-03

**Authors:** Haonan Lin, Liming Shang, Yinping Lu

**Affiliations:** 1 Department of Otolaryngology, Affiliated Hospital of Shaoxing University, Shaoxing, China; 2 Department of Otorhinolaryngology, Shaoxing People’s Hospital, Shaoxing, China

**Keywords:** microecological intervention, postoperative recurrence, sinus microbiome, type 2 chronic rhinosinusitis with nasal polyps, type 2 inflammation

## Abstract

The repetition of a condition following endoscopic sinus surgery (ESS) poses an important difficulty in type 2 chronic rhinosinusitis with nasal polyps (CRSwNP) therapy. The recurrent nature of the disease is probably linked to the dynamic interplay of the enduring type 2 inflammatory microenvironment and the imbalanced sinonasal microbiome. The microbiomic characteristics and pathogenic mechanisms of postoperative recurrence are systematically elaborated in this paper, indicating that recurrent CRSwNP is characterized by microbial dysbiosis featured by enrichment of Staphylococcus aureus, depletion of Corynebacterium, and reduction in α-diversity. Its core mechanisms involve superantigen-mediated immune hijacking, epithelial barrier collapse caused by microbial metabolic imbalance, and physiological tolerance mediated by biofilms. On this basis, as a scoping review, this paper further reviews the shift of intervention strategies from “broad-spectrum bactericidal” to “precise ecological restoration”, including narrow-spectrum antibacterial and phage therapies targeting Staphylococcus aureus, ecological replacement therapy by supplementing nasal-specific commensal bacteria (such as Corynebacterium), and synergistic regulation strategies using biological agents such as dupilumab to indirectly reshape the microecology by inhibiting type 2 inflammation. In addition, the paper also discusses the potential of constructing postoperative recurrence prediction models based on microbial biomarkers, aiming to provide a theoretical basis for the precise and dynamic management of CRSwNP.

## 1 Introduction

Recurrent type 2 chronic rhinosinusitis(CRS)with nasal polyps (CRSwNP) following endoscopic sinus surgery (ESS) presents a significant clinical obstacle, marked by ongoing mucosal inflammation and a pronounced likelihood of polyp regrowth (1). The recurrence of nasal polyps post-surgery is notably frequent. Research indicates that approximately 35% to 40% of patients experience recurrence within 18 months after the procedure (2), while long-term studies reveal that this rate can soar to 78.9% over a span of 12 years (3). To evaluate recurrence, clinicians utilize the Lund Kennedy score and various endoscopic scoring systems that assess polyp size, edema, and the emergence or worsening of symptoms like nasal blockage and loss of smell (4). Additionally, established risk factors for recurrence encompass a history of prior surgeries, severe preoperative polyposis, and associated conditions such as asthma and aspirin-exacerbated respiratory issues. A higher number of affected sinuses correlates with a more severe disease state, thereby elevating the risk of recurrence postoperatively (5).

The type 2 inflammatory endotype is a significant underlying cause of CRSwNP, contributing to its high recurrence rates in both China and Western nations (4, 6). This inflammatory condition is marked by an excessive production of cytokines such as interleukin IL-4, IL-5, and IL-13, which facilitate eosinophil infiltration, local IgE synthesis, and tissue remodeling (7). Eosinophils serve as the primary effector cells in this process, and their levels in tissue can indicate the likelihood of recurrence following surgical intervention. They are also a major factor in eosinophilic esophagitis. Research has shown that a tissue eosinophil threshold of 27% in Chinese patients with CRSwNP can predict recurrence with a sensitivity of 96.7% and specificity of 92.5% (4). Type 2 inflammation persists after surgery, as evidenced by significant increases in serum levels of IL-13, eotaxin, RANTES, IL-17A, and other related factors in recurrent patients. This ongoing type 2 inflammation and tissue remodeling create a microenvironment conducive to polyp formation (8). Additionally, refractory cases, which require revision surgery despite optimal medical treatment, may exhibit an increase in Th17/Th1-related mediators, indicating a potential shift from a primarily eosinophilic inflammatory profile, suggesting that the endotype may evolve over time or with successive procedures (1).

Consequently, approaches to manage ongoing and stubborn inflammation must extend beyond merely removing polyps. Currently, the available methods to avert recurrence have their drawbacks. Typically, post-operative medical care involves administering both topical and systemic glucocorticoids to mitigate inflammation, along with antibiotics to address possible infections (9). However, these treatment plans often fail to ensure sustained control of the disease, with a notably high rate of recurrence, especially among individuals experiencing severe type 2 inflammation (1). The advent of the biologic medication dupilumab presents a promising and more precise way to modulate the fundamental type 2 inflammatory pathway. Research indicates that dupilumab can effectively decrease polyp size, enhance symptoms, and reduce reliance on systemic steroids and surgical interventions (10).

Key biomarkers, such as the count of blood eosinophils, play a crucial role in selecting patients for biologic therapies aimed at identifying those with type 2 inflammatory characteristics (11). Additionally, the patient’s microbiome has emerged as a significant element influencing both treatment effectiveness and the likelihood of recurrence. CRSwNP is closely associated with an imbalance in sinonasal microbial communities, characterized by a loss of healthy microbial diversity and an increase in opportunistic pathogens like Staphylococcus aureus. This microbial imbalance perpetuates type 2 inflammation, as research indicates that Staphylococcus aureus can trigger the release of cytokines from epithelial cells, such as IL-33, Thymic Stromal Lymphopoietin, and IL-25, which in turn enhance Th2 immune responses (12). Moreover, the interplay between ongoing type 2 inflammation and a disrupted microbiome may create a self-perpetuating cycle that diminishes the effectiveness of standard treatments and biologics. Thus, there is an urgent need to deepen our understanding of the microbiological landscape following surgery to enhance long-term patient outcomes.

## 2 The sinus microbiota: methodologies and key quality control points

Investigating the sinus microbiome is crucial for understanding the development of CRS. Nonetheless, various limitations, including the nature of samples, the techniques used for analysis, and the clinical contexts, can lead to significant biases in both study design and the interpretation of findings. The primary challenges related to techniques and methodologies are evident in three key areas.

### 2.1 Core technical challenges: low biomass and pollution control

Sinus samples exhibit a notably reduced bacterial biomass compared to oral and other locations, a difference that is especially evident in healthy subjects. These samples are particularly vulnerable to external contamination, which significantly skews the results (13, 14). The primary sources of this contamination include bacteria from reagents and those introduced by the surrounding environment. Implementing negative controls at every stage of the process is essential for accurately differentiating true microbial signals from background interference (13).

### 2.2 Misunderstandings in data interpretation: limitations of diversity and relative abundance

The alpha diversity of microbiota in CRS serves as a measure of the variety and distribution of species within an individual sample. In certain CRS phenotypes, particularly those without nasal polyps, there is a notable reduction in bacterial diversity when compared to healthy individuals. Conversely, phenotypes like CRSwNP do not exhibit these variations. It is essential to analyze these discrepancies alongside inflammatory endotypes (13, 15). Many research efforts rely solely on relative abundance metrics, which fail to accurately represent the actual presence of harmful bacteria; therefore, methods like quantitative real-time polymerase chain reaction are necessary to assess critical bacteria such as Staphylococcus aureus.

### 2.3 Key confounding factors: clinical interventions and individual differences

Therapeutic measures can profoundly influence the composition of the microbiome: the use of antibiotics diminishes diversity and promotes the emergence of resistant bacterial strains, glucocorticoids modify the immune landscape, and endoscopic sinus surgery changes the conditions for microbial colonization, each having distinct impacts. Variations in microbiota linked to individual host factors (such as age, asthma, past surgeries, and allergies) frequently surpass the differences observed between healthy and diseased populations, and analyses that are not adjusted may lead to misleading positive findings (14–16).

A range of methodological shortcomings undermines the reliability of current findings regarding the sinus microbiome in recurrent CRSwNP. To begin with, the lack of adequate negative controls for low-biomass sinus samples can easily result in background contamination, which in turn leads to variable microbial profiles among different groups. Additionally, many studies focus exclusively on relative abundance instead of absolute quantification, complicating the ability to differentiate between genuine pathogen proliferation and the loss of commensal organisms. Furthermore, various clinical factors, including surgical history, medication use, and allergic conditions, are often not thoroughly accounted for, potentially creating misleading associations between dysbiosis and the recurrence of disease. In summary, these technical issues contribute to significant variability between studies; the existing correlational data do not provide a solid basis for causal inferences, highlighting the urgent need for standardized methodologies in future investigations.

## 3 Microbiomic characteristics of recurrent type 2 CRS with nasal polyps

The diversity of the microbiome in chronic rhinosinusitis plays a vital role in categorizing the disease and assessing its prognosis. Research has classified CRS patients into various subgroups based on their microbial community structures, indicating that those primarily influenced by Corynebacteriaceae and Staphylococcaceae show notable differences in clinical features, such as immune responses and the occurrence of nasal polyps (17). This insight offers theoretical guidance for understanding the microecological mechanisms behind recurrent type 2 CRSwNP (**Table 1**).

**Table 1:** Microbiome characteristics and clinical phenotypes of recurrent CRSwNP.

Evaluation indicators	Changes in key flora/indicators	Clinical phenotype and significance
Typing of main flora subgroups	Dominated by Corynebacteriaceae and Staphylococcaceae	There are significant differences in host immune responses and the incidence of nasal polyps among different dominant subsets.
Community composition at the phylum level	The abundance of Actinomycetes decreased; the abundance of Proteobacteria and Firmicutes increased.	Suggesting a fundamental restructuring of the nasal microbiome
Pathogenic bacteria at the genus/species level	Increased abundance of Staphylococcus (especially Staphylococcus aureus)	It is closely related to the severity and refractoriness of the disease, and is particularly prominent in NECRSwNP.
Potential protective bacteria	Decreased abundance of Corynebacterium	Its presence is associated with a favorable postoperative prognosis; the shift in the flora structure from Corynebacterium to Staphylococcus is a key feature of recurrence.
α-diversity	Reduce	It is more prominent in the high recurrence risk subtype, but there is certain heterogeneity among different studies.
β-diversity	Significantly increased	It suggests that the inter-individual differences in the microbiota increase, homeostasis is disrupted, and a variety of recurrent dysbiotic phenotypes emerge.

CRSwNP, chronic rhinosinusitis with nasal polyps; NECRSwNP, non-eosinophilic chronic rhinosinusitis with nasal polyps.

The microbiome profile of recurrent type 2 CRSwNP exhibits distinct taxonomic features. At the phylum level, there is a notable decline in Actinomycetes alongside a rise in Proteobacteria and Firmicutes, coupled with a marked reduction in beneficial bacteria such as Corynebacterium, suggesting a significant reorganization of the nasal microecosystem (18).

Dysbiosis of the microbiome is particularly evident at the genus and species tiers. In individuals suffering from recurrent CRSwNP, there is a notable increase in the presence of Staphylococcus, especially Staphylococcus aureus, which is especially marked in those with the non-eosinophilic phenotype. The level of this bacterium is closely linked to the severity of the disease and the refractory nature of the condition (19, 20).

In contrast, the occurrence of Corynebacterium is generally diminished in cases of recurrence, and its presence is associated with a better postoperative outcome, implying a possible protective function (15, 20). However, existing animal models designed to establish a causal relationship between Staphylococcus aureus and CRS have notable limitations. Most traditional murine models of CRS induced by Staphylococcus aureus use specific pathogen-free (SPF) mice that harbor native commensal bacteria, which complicates the elimination of microbial confounding variables; there are no reports of standardized germ-free mouse models that are intranasally colonized with Staphylococcus aureus. Additionally, significant pathological differences are observed between current CRS animal models and human conditions, resulting in variable inflammatory responses to pathogens across different species, which hampers the ability to translate animal research into clinical relevance (21, 22). Nonetheless, the alteration in microbial ecology, indicated by an increased ratio of Staphylococcus to Corynebacterium, may signify a more severe or enduring inflammatory state. The shift from a Corynebacterium-dominant to a Staphylococcus-dominant microbial community is a notable characteristic of the recurrent CRSwNP microbiome (23). The diversity of the community serves as an effective marker to differentiate between recurrent CRSwNP, non-recurrent cases, and healthy individuals. Patients with recurrent conditions show lower α-diversity, characterized by a less complex microbial structure and reduced stability, particularly in high-risk endotypes for recurrence, although variability among studies exists. The β-diversity, which assesses microbial differences among samples, is markedly elevated, reflecting increased variation among individuals, disrupted microbial balance, and the emergence of various dysbiotic phenotypes that are susceptible to recurrence (19, 20).

Research on microbiomes reveals both consistent patterns and significant variability, shaped by various confounding elements. One primary factor contributing to this variation is geographical location, which leads to notable differences in the prevalence of disease endotypes, environmental factors, and the composition of host microbiomes among different populations, such as those in Europe and Asia (19, 20, 24). Prior endoscopic sinus surgeries can modify sinonasal structures, influencing subsequent microbial colonization; longitudinal research indicates a postoperative transition in dominant bacterial species from Streptococcus to Staphylococcus. The severity of disease, measured by tools like the Sino-Nasal Outcome Test-22 or Lund-Mackay scores, correlates with the presence of certain microbiota, where higher levels of Escherichia coli and Dialister are linked to increased symptom severity (20). Additionally, the methods of sampling—whether using swabs or tissue samples, and whether taking single or multiple sinus samples—can introduce discrepancies, as microbial profiles can vary between sinuses within the same individual. These considerations are crucial for accurately interpreting and comparing findings across different studies (14).

## 4 Recurrence mechanisms of core pathogenic bacteria and microbial pathways

### 4.1 Immune hijacking: abnormal immune activation mediated by superantigens

Staphylococcus aureus produces staphylococcal enterotoxins (SEs), which are strong proinflammatory superantigens capable of triggering severe type 2 inflammatory reactions in the nasal mucosa. In clinical settings, higher concentrations of SE-specific IgE (SE-IgE) correlate significantly with the advancement of the disease and the recurrence of polyps in chronic rhinosinusitis with nasal polyps (CRSwNP). This indicates that SEs might play a crucial role in the recurring nature of CRSwNP. Nonetheless, additional clinical studies are required to confirm a direct relationship between these factors (25, 26).

SEs bypass the traditional processing typically needed by antigen-presenting cells; rather, they can directly connect Major Histocompatibility Complex class II molecules to the beta variable region of the T cell receptor, leading to the nonspecific activation of a significant population of T cells (27, 28).

Activated T cells extensively secrete Th2 cytokines, including IL4, IL5, and IL13, which stimulate B cells to generate SEs-specific IgE (6).This IgE has the potential to sensitize mast cells, leading to swift degranulation and perpetuating chronic inflammation and tissue remodeling (29).

**Figure 1:**
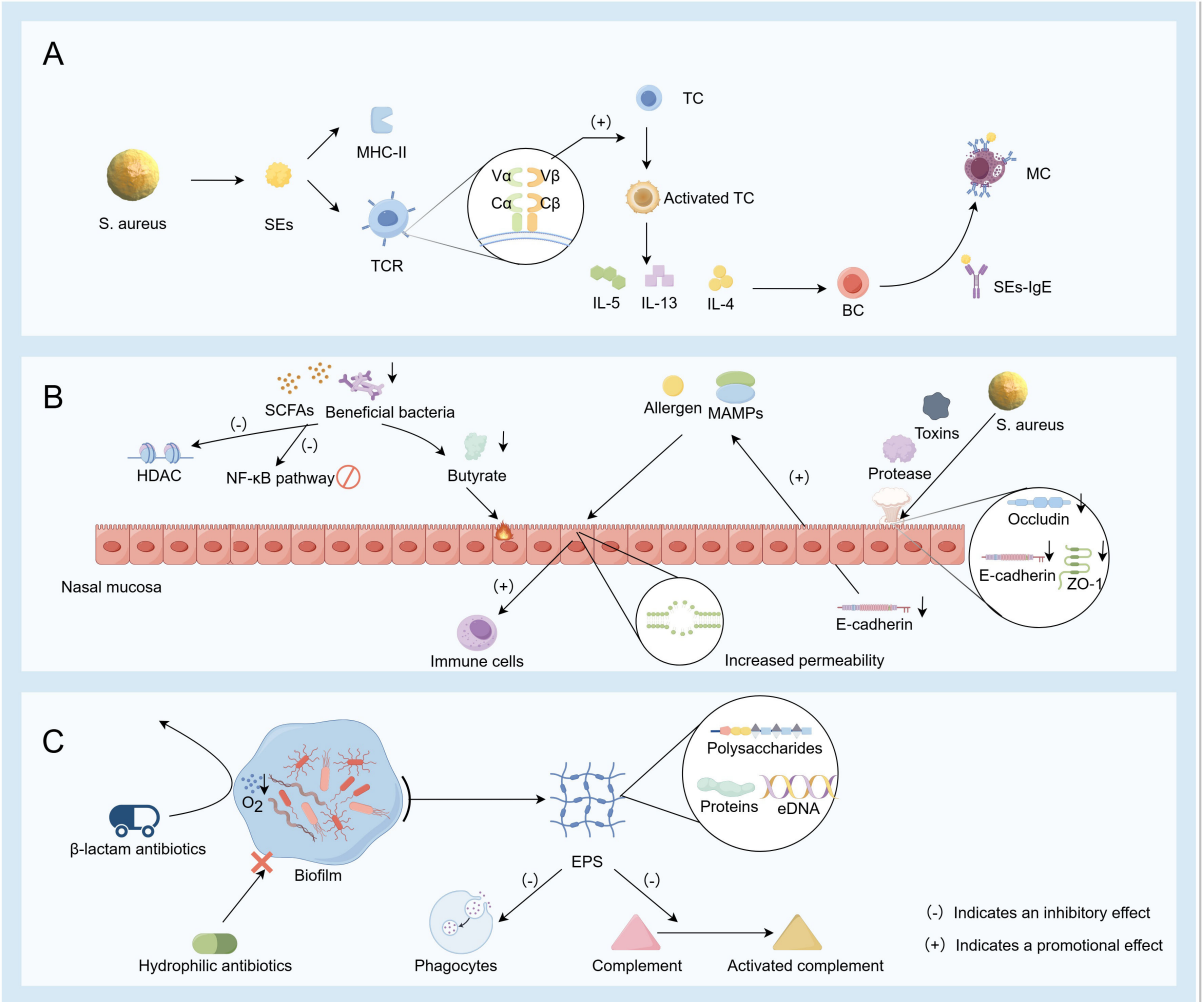
Mechanisms of disease recurrence mediated by core pathogenic bacteria and microbial pathways. **(A)** Staphylococcus aureus secretes SEs that act as superantigens. SEs directly crosslink MHC-II and TCR Vβ to non-specifically activate T cells, triggering Th2 cytokines (IL-4, IL-5, IL-13) and SE-specific IgE production. **(B)** Microbiota dysfunction disrupts the nasal mucosal barrier and activates immunity, forming a vicious cycle with pathogen colonization. S. aureus toxins impair tight junctions while reduced butyrate hinders repair and activates NF-κB, aggravating type 2 inflammation in recurrent CRSwNP. **(C)** The physical structure of biofilms blocks hydrophilic antibiotics, and intramembrane hypoxia induces dormant persister cells to evade β-lactams. EPS inhibit phagocytosis and complement activation, combined with Th2 inflammation driven by S. aureus superantigens and mucosal barrier damage caused by microbial dysfunction. S. aureus, Staphylococcus aureus; SEs, Staphylococcal enterotoxins; MHC-II, Major Histocompatibility Complex class II; TCR, T-cell receptor; TCR Vβ, T cell receptor beta variable region; TC, T cell; BC, B cell; SEs-igE, Staphylococcal enterotoxin-specific IgE; MC, Mast cell; HDAC, Histone deacetylase; SCFAs, Short-chain fatty acids; ZO-1, Zonula Occludens-1; EPS, Extracellular polymeric substances.

In Western nations, elevated levels of IL5 facilitate the recruitment, activation, and longevity of eosinophils, which contribute to the characteristic pathological traits of CRSwNP. This phenomenon elucidates how IL5 significantly enhances the type 2 inflammatory environment by promoting eosinophil activity and survival, leading to increased local IgE concentrations in individuals without allergies (6). These mechanisms are depicted in **Figure 1A**. Nonetheless, the activation of type 2 inflammation does not solely rely on SEs. Research involving clinical cohorts has shown that some patients suffering from recurrent CRSwNP do not express SEs yet still exhibit pronounced type 2 inflammation, thereby confirming the presence of pathogenic mechanisms that operate independently of SEs (30, 31). Further mechanistic investigations indicate that the SE-dependent and SE-independent pathways are seldom activated at high levels simultaneously, suggesting a dynamic interplay where one pathway may intensify while the other diminishes (32).

### 4.2 Barrier collapse: microbial metabolic imbalance and epithelial transformation

Impairment of the microbiome triggers immune reactions and simultaneously compromises the structural integrity of the nasal mucosa. This results in a worsening pathological cycle where damage to the barrier and the colonization by harmful bacteria intensify one another (33). The mechanisms behind this barrier compromise and the resulting inflammation primarily involve two processes: the deterioration of tight junctions and an imbalance in microbial metabolism.

Disruption of tight junctions occurs when toxins and proteases released by harmful bacteria like Staphylococcus aureus target and break down tight junction proteins such as Occludin and Zonula Occludens1, along with the adhesion protein Ecadherin. A decrease in Ecadherin levels compromises the integrity of the epithelium and heightens mucosal permeability, enabling Microbe-Associated Molecular Patterns and allergens to infiltrate the epithelial barrier and stimulate immune responses, which exacerbates inflammation (34, 35).

Metabolite disruption: In individuals suffering from recurrent CRSwNP, there is a notable decrease in beneficial bacteria that generate short-chain fatty acids, leading to lower concentrations of anti-inflammatory metabolites like butyrate (36). This reduction not only compromises the energy supply to epithelial cells and obstructs barrier repair but also instigates excessive inflammatory reactions by lifting the suppression of the NF-κB pathway and diminishing Histone Deacetylase activity (37–39). The intricate mechanisms involved are illustrated in **Figure 1B**. Nonetheless, the existing evidence supporting butyrate’s protective role in mucosal health primarily comes from *in vitro* studies and animal research (40, 41), with a significant gap in consistent clinical data from humans that verifies the effective anti-inflammatory concentration of topically applied butyrate in the nasal cavity. This phenomenon of barrier disruption is further elucidated at the regulatory pathway level: the IL-4/IL-13 pathway, influenced by the upstream transcription factor NF-κB, shows a reciprocal interaction, where both pathways can inversely affect epithelial integrity, potentially leading to antagonistic outcomes (42).

### 4.3 Survival fortress: the dual tolerance mechanism of biofilms

The development of biofilms plays a crucial role in the recurrence and resistance seen in CRSwNP, as they significantly bolster bacterial evasion of the immune system and resistance to antibiotics (43). These biofilms create compact three-dimensional formations that depend on extracellular polymeric substances, which include polysaccharides, proteins, and extracellular DNA. This structure acts as a physical barrier, impeding the entry of hydrophilic antibiotics and preventing effective drug concentrations from reaching the infected areas (44). Additionally, the low oxygen levels and nutrient scarcity within biofilms slow bacterial metabolism, allowing persister cells to enter a dormant or low-activity state, thus escaping the effects of β-lactam antibiotics that target actively dividing bacteria (45, 46). Furthermore, the extracellular polymeric substance(EPS) matrix can obstruct phagocytosis by immune cells and disrupt complement activation, resulting in prolonged immune responses and tissue damage without fully eradicating the encapsulated bacteria (47). The mechanisms behind recurrent CRSwNP involve complex molecular interactions: superantigens from Staphylococcus aureus improperly activate the immune response, leading to Th2-type inflammation and local IgE production, while microbial metabolic disturbances compromise the nasal mucosal barrier, and biofilm formation offers ongoing protection for pathogenic bacteria (37, 48, 49). The interplay of these three elements constitutes the fundamental pathophysiological framework for chronic disease and postoperative recurrence. These interconnected processes are illustrated in **Figure 1C**. However, the relationship between biofilm formation and toxin secretion, including superantigens, is not merely synergistic. Research has shown that these two processes are dynamically regulated through the Agr quorum-sensing system: during the initial phase of biofilm formation, agr system activity is suppressed, facilitating bacterial clustering and extracellular matrix production; conversely, in the later stages of biofilm maturation or dispersal, the agr system becomes active, leading to increased expression of virulence factors like superantigens and promoting biofilm disassembly. Previous literature has often oversimplified this interaction as a straightforward synergistic effect, neglecting the complex, stage-dependent regulatory mechanisms that exist between biofilm and toxin pathways (50).

## 5 Microbiome characteristics and recurrence prediction model

The essence of upcoming studies focuses on converting microbiome information into tools for clinical decision-making, facilitating a transition in CRSwNP from a reactive approach to a more proactive strategy.

**Figure 2:**
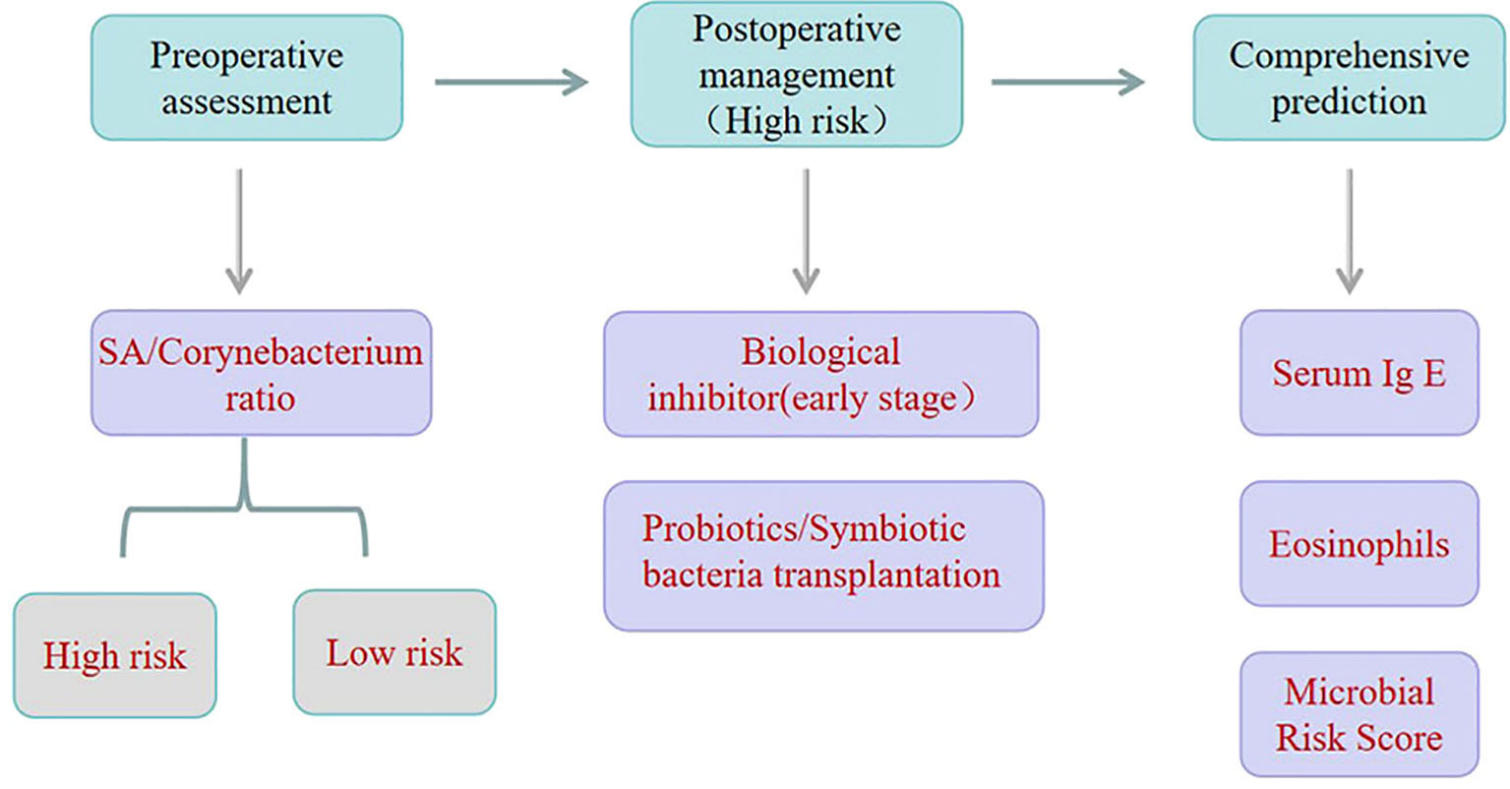
Potential decision-making process for CRSwNP (process schematic diagram). Preoperatively, high and low risks are assessed according to the SA/Corynebacterium ratio. For patients at high risk, early intervention with biological inhibitors and probiotics or symbiotic bacteria is administered to mitigate the risk. In the future, serum IgE, eosinophil count, and microbial scores can be integrated to predict postoperative recurrence. CRSwNP, chronic rhinosinusitis with nasal polyps; SA, Staphylococcus aureus.

Before surgery, a high ratio of Staphylococcus aureus to Corynebacterium can help pinpoint patients at elevated risk. Nonetheless, this ratio has not yet been validated in prospective studies, lacks reproducibility, and does not have standardized protocols across different research, so it should be viewed as a potential biomarker rather than a confirmed prognostic tool. A meta-analysis involving nine randomized controlled trials and 1,146 patients demonstrated that the use of multi-strain probiotics during the perioperative period significantly lowered the incidence of overall postoperative infections, surgical site infections, and non-surgical site infections in colorectal procedures, while single- or dual-strain probiotics did not show a notable protective effect (51). Consequently, patients may benefit from antibiotic irrigation, topical antimicrobial peptides, or brief pretreatment with biological agents targeting type 2 inflammation to restore microecological balance. After surgery, high-risk patients undergo rigorous management, which includes more frequent endoscopic debridement and early introduction of probiotics or transplantation of commensal microbiota. Research in liver transplantation has shown that the combined postoperative use of multi-strain probiotics, including Lactobacillus and Bifidobacterium, decreased the bacterial infection rate from 30.3% to 8.8%, significantly reducing infections and shortening the length of antibiotic treatment (52). In the future, inflammatory markers like serum IgE and eosinophil counts in peripheral blood could be integrated with microbial risk assessments to forecast the recurrence of CRSwNP after surgery (20, 53, 54). Incorporating anatomical characteristics will facilitate the development of a visual decision-making tool, enhance the application of rapid polymerase chain reaction detection, and establish a precise system for diagnosis and treatment to inform surgical timing, the use of biological agents, and secondary interventions (**Figure 2**).

While the previously mentioned diagnostic and treatment framework shows encouraging potential for application in clinical settings, significant debates and clear gaps in evidence persist within the current landscape. Firstly, research has yet to clarify whether the initial preoperative microbiota or the evolving postoperative microbiota of CRSwNP offers greater predictive accuracy for recurrence. This unresolved issue highlights a possible competitive dynamic between these two microbial biomarkers. Additionally, the expensive nature and lengthy processing times of microbial sequencing tests hinder their ability to fulfill the urgent requirements for swift clinical assessments (55); concurrently, many existing predictive models are still in the theoretical phase, lacking robust prospective interventional studies to validate their effectiveness in guiding clinical choices and minimizing postoperative recurrence rates, leading to an incomplete chain of evidence (56).

## 6 Targeted precise intervention of the microbiome

Currently, the primary treatment approach for CRSwNP involves the use of ESS alongside systemic glucocorticoids. Recently, there has been a growing focus on the targeted manipulation of the nasal microbiome, either directly or indirectly, as a promising strategy to lower the chances of recurrence after surgery. Conventional broad-spectrum antibiotics often disturb the delicate balance of the nasal microecosystem and can lead to the development of bacterial resistance. As a result, modern therapeutic strategies prioritize precise interventions that aim to eradicate harmful bacteria while preserving beneficial microbial communities, thereby fostering a stable nasal environment (**Table 2**).

**Table 2:** Comparison table of advantages and disadvantages of existing nasal microecological intervention programs.

Intervention strategies	Representative plan	Target of action	Advantages	Limitations	Clinical translation stage
Narrow-spectrum antimicrobials/Bacteriophages	Phage Cocktail APTC-C-SA01	Staphylococcus aureus, biofilm	Precise sterilization without disrupting commensal bacteria	High costs and difficulties in individualized customization	*In vitro*/small-sample clinical trials
Auxiliary biofilm dissolution	N-Acetylcysteine	Degradation of biofilm matrix	It exhibits a significant effect against biofilms *in vitro*.	It is difficult to achieve an effective concentration in the body	*In vitro* basic research
Symbiotic bacteria replacement therapy	Corynebacterium, Lactococcus lactis W136	Reconstruct the beneficial flora in the nasal cavity	Long-term reshaping of the micro-ecosystem with high safety	Strong strain specificity and unstable colonization efficiency	*In vitro* cell/strain experiments
Indirect regulation of biological agents	Dupilumab	Inhibit type 2 inflammation and indirectly repair the flora	Approved for marketing with sufficient clinical evidence	Cannot directly eliminate pathogenic bacteria	Mature clinical application
SinoNasal Microbiota Transfer	Transplantation of nasal commensal flora from healthy donors	Correct nasal flora imbalance, regulate microecology, and alleviate inflammation and related symptoms	It significantly alleviates the symptoms of refractory CRSwNP and has favorable short-term safety.	Insufficient evidence-based evidence and obvious biases in research design, among other factors.	Clinical exploration stage

CRSwNP, Chronic rhinosinusitis with nasal polyps;

### 6.1 Precise antibacterial action and phage targeted therapy

While conventional broad-spectrum antibiotics, whether systemic or topical, are designed to eradicate harmful pathogens, they often disturb the natural microbial community and contribute to the development of drug resistance. Additionally, these antibiotics are generally not effective against infections linked to biofilms in cases of recurrent CRSwNP (57, 58). Due to the considerable variability in the microbiomes of patients, there is a need for clinical approaches to become more precise. Conducting metagenomic analysis before surgical procedures or before the onset of recurrence can help identify a patient’s unique microbial fingerprint, facilitating tailored therapeutic strategies.

Infections dominated by Staphylococcus aureus can be effectively treated with narrow-spectrum antibiotics or targeted phage therapy, which can specifically eliminate harmful bacteria, infiltrate biofilms, and maintain beneficial microbial communities. The phage mixture APTCCSA01, created in 2022, is designed to specifically attack biofilms formed by Staphylococcus aureus and Methicillin-resistant Staphylococcus aureus, offering a new method for addressing infections linked to biofilms (59). As an innovative precision antibacterial technique, phage therapy allows for targeted elimination of pathogens while preserving the natural flora due to its remarkable host specificity. Nevertheless, the application of this approach in clinical settings encounters several obstacles. The expenses associated with metagenomic sequencing and personalized treatment plans, such as custom phages, are quite high, and the long-term advantages compared to traditional treatments (like biologics and surgical options) have yet to be validated through health economic research (60). Furthermore, there are two significant gaps in clinical practice: there are no standardized protocols for both systemic and localized phage therapy administration, and there is a lack of large-scale randomized controlled trials to demonstrate its effectiveness in reducing infection recurrence over time (61, 62).

Patients lacking adequate commensal flora can benefit from the addition of targeted strains to help reestablish their microecological equilibrium. Research has demonstrated that intranasal irrigation using live Lactococcus lactis W136 is both safe and beneficial for individuals with persistent CRS who have had prior surgical interventions, leading to enhancements in symptoms, mucosal health, and the composition of the microbiome (63).

The impact of probiotics varies significantly between different strains. Research conducted *in vitro* indicates that Lactococcus lactis W136 has contrasting effects on Pseudomonas aeruginosa: it suppresses the growth of certain strains, enhances the growth of mucoid strains, and shows negligible effects on others, highlighting its distinct strain specificity and reliance on pathogenic characteristics (64).

In cases of mixed infections, employing multimodal approaches alongside strategies for biofilm removal can be beneficial. A study conducted in 2025 demonstrated that N-acetylcysteine (NAC) could significantly inhibit and eliminate Staphylococcus aureus biofilms in patients with CRS at specific concentrations (65). However, there is often a discrepancy between laboratory results and real-world effectiveness, as achieving the necessary concentrations in human subjects is challenging. Research indicates that NAC can completely prevent staphylococcal biofilm formation at a concentration of 3.1 mg/mL, which is half of the Minimum Inhibitory Concentration (MIC). To eradicate established biofilms, a much higher dosage is required—up to 49.6 mg/mL, which is eight times the MIC. Furthermore, elevated levels of NAC may diminish the efficacy of carbapenem antibiotics, and such high concentrations are typically not reached in human patients (66).

### 6.2 Ecological replacement and microecological restoration

Merely sterilizing does not address the underlying causes of disease recurrence. The focus of treatment has evolved towards reconstructing a robust microbial community that can resist pathogen invasion, aiming for sustainable ecological recovery. Recent studies have concentrated on commensal bacteria specific to the nasal environment. Corynebacterium pseudodiphtheriticum is a key commensal organism found in a healthy nasal cavity, capable of inhibiting Staphylococcus aureus growth through competitive displacement and the release of antimicrobial compounds (67). Corynebacterial strains such as Corynebacterium accolens are highly abundant in the nasal cavity of healthy individuals but significantly reduced in patients with CRSwNP. Their culture supernatants can effectively inhibit the proliferation of Staphylococcus aureus and delay mucosal barrier damage, showing promising potential as high-quality probiotics (68). Strains like Corynebacterium accolens are prevalent in the nasal cavities of healthy individuals but are notably diminished in those with CRSwNP. Their culture supernatants have been shown to effectively suppress Staphylococcus aureus proliferation and mitigate damage to the mucosal barrier, indicating their potential as effective probiotics. Beyond direct supplementation of these strains, enhancing the growth of beneficial nasal bacteria with targeted metabolic substrates, fostering their establishment, and restoring microbial diversity can serve as a gentle and enduring ecological restoration approach to collaboratively establish a protective microbial barrier. Research has demonstrated that Corynebacterium accolens can break down host triacylglycerols through the LipS1 lipase, releasing antibacterial free fatty acids that inhibit the colonization of pathogens like Streptococcus pneumoniae. This suggests that preserving beneficial commensal bacteria, such as Corynebacterium, is crucial for defending against respiratory pathogens and maintaining the balance of the nasal microecology (69). However, current interventions targeting the nasal microbiome with Corynebacterium face notable limitations. Firstly, these bacterial strains are highly specific; the antibacterial properties of one strain do not guarantee similar effects from other Corynebacterium strains (70). Secondly, research has shown that the retention of Corynebacterium after a single treatment is inadequate, making it challenging to achieve a stable, long-term remodeling of the nasal microbiome through just one instance of microbial flora supplementation (71).

### 6.3 Synergistic regulation of the immune-microbiota axis

The fundamental issue in recurrent CRSwNP lies in the disrupted interaction between the host’s immune system and the microbiome, where type 2 inflammation serves as the primary trigger and dysbiosis results as a secondary effect. Elevated levels of IL4 and IL13 can directly compromise the integrity of the nasal mucosal barrier (17), modify mucus characteristics (72), and diminish the synthesis of antimicrobial peptides (73). This situation fosters the growth and establishment of harmful bacteria like Staphylococcus aureus (42, 74), creating a detrimental cycle of “inflammation-dysbiosis-persistent infection”.

Biological therapies like dupilumab offer a novel approach to indirectly modulate the microbiota. By inhibiting the IL4/IL13 signaling pathway, this medication not only reduces eosinophilic inflammation but also aids in restoring the epithelial barrier (75), enhances mucus production (76), and improves the microenvironment’s physicochemical properties (77). This action helps control the overgrowth of harmful bacteria and fosters the reestablishment of microbiota balance (78), effectively addressing the cycle of “inflammation-dysbiosis”. Additionally, patients with CRS can be stratified by integrating microbiome data with inflammatory indicators. Identifying high-risk individuals through their nasal microbial profiles—characterized by a high presence of Staphylococcus aureus and a low presence of Corynebacterium—can lead to timely administration of biological treatments, adjustments in therapy, and monitoring of treatment effectiveness. Customized interventions based on microbial endotypes can prevent ineffective treatments and allow for focused strategies. Nonetheless, the impact of these drugs on nasal microbiota remains a topic of clinical debate. Research indicates that a 24-week treatment with dupilumab did not significantly change the overall nasal microbiome structure (79), implying that managing inflammation and repairing nasal flora is not merely a straightforward cause-and-effect scenario. Other factors, such as antibiotic use, anatomical issues in the sinuses, and various immune pathways, can perpetuate dysbiosis, indicating that relying solely on this biological therapy may not fully rectify the underlying microecological imbalances.

### 6.4 SinoNasal microbiota transfer

SinoNasal Microbiota Transfer (SNMT) is a novel therapeutic approach that shares similarities with fecal and oral microbiota transplants. This method involves transferring nasal flora from healthy individuals to patients in order to restore the balance of nasal microbiota, potentially enhancing outcomes for respiratory conditions like CRS (80, 81). Initial findings from a small clinical case series suggest that nasal microbiota transplantation may be beneficial for patients with persistent chronic rhinosinusitis, as those who underwent the procedure showed a temporary boost in nasal microbiota diversity, with some experiencing lasting improvements in their Sinonasal Outcome Test-22 quality of life scores (82). Additionally, studies focusing on refractory CRSwNP have indicated that all participants in the SNMT treatment group reported relief from both endoscopic and subjective symptoms shortly after the procedure, with two-thirds maintaining this improvement for up to 180 days, and no significant adverse effects were noted, supporting the short-term safety and effectiveness of this treatment (83).

While current research has tentatively established the short-term effectiveness and potential use of SNMT, this treatment still faces several significant limitations. Firstly, there is a scarcity of robust evidence; no extensive randomized double-blind studies have validated its long-term effects on preventing recurrence, and the link between microbiota enhancement from transplantation and the reduction of inflammation is still not well understood (84). Secondly, there are notable flaws in the research methodologies. Non-randomized open-label studies are susceptible to biases and placebo effects. Additionally, variations in antibiotic pretreatment, inconsistent transplantation methods, and differing evaluation standards hinder the ability to make direct comparisons across studies, leading to a lack of reliable evidence (82, 84). Moreover, the therapy requires complex donor screening processes, relies on endoscopic equipment for transplantation, and incurs relatively high costs for each intervention, which currently limits its widespread application.

## 7 Conclusion and prospects

Recent studies on the sinus microbiome in patients with CRSwNP have shifted from merely identifying correlations to investigating underlying mechanisms. The recurrent nature of type 2 CRSwNP is fueled by a self-perpetuating cycle characterized by ongoing type 2 inflammation and imbalances in sinonasal microbial communities, with the sinus microbiome playing a crucial role in recurrence, resistance to treatment, and overall disease outlook. The Staphylococcus aureus to Corynebacterium ratio has emerged as a potential clinical indicator. Contemporary treatment approaches have evolved from using broad-spectrum antibiotics to more integrated methods that focus on bactericidal actions, mucosal healing, and ecological balance, incorporating strategies like targeted phage therapy, Corynebacterium-based nasal probiotics, and tailored dupilumab treatments. However, several translational challenges persist: the effectiveness of Corynebacterium probiotics is hindered by strain specificity and limited nasal retention; phage therapies face high customization costs and inconsistent administration methods; and dupilumab does not address dysbiosis caused by anatomical changes or antibiotics, failing to significantly alter nasal microbiota in short-term studies. Consequently, future research should prioritize three main areas: confirming the predictive power of microbial biomarkers through extensive long-term studies, enhancing mucosal delivery systems for sustained release of phages and beneficial microbes, and developing robust nasal probiotic strains. By integrating multi-omics analyses of sinonasal microbiota and inflammatory factors, future investigations can create a comprehensive microecology-guided precision framework for preoperative risk assessment and ongoing postoperative monitoring, ultimately aiming to reduce the rate of recurrence following ESS.
